# Connected by Nature: Nature-Based Intervention to Support Young Adults with Advanced Cancer

**DOI:** 10.3390/ijerph23080953

**Published:** 2026-07-24

**Authors:** Sus Sola Corazon, Maria Stoltze Fray, Gitte Saldern Schroeder, Carina Wedell Andersen, Stine Bekke-Hansen

**Affiliations:** 1 Department of Geosciences and Natural Resource Management, University of Copenhagen, 1958 Frederiksberg C, Denmark; 2 Copenhagen Centre for Cancer and Health, 2200 Copenhagen N, Denmark; fq8i@kk.dk (M.S.F.); ed7d@kk.dk (C.W.A.); 3 Network for Adolescents and Young Adults Cancer Research in Denmark (NAYAcare DK), Danish Comprehensive Cancer Center (DCCC), 8200 Aarhus, Denmark

**Keywords:** rehabilitation, palliation, rehabilitative palliation, nature-based health care, nature intervention, existential health, chronic cancer, non-curable cancer

## Abstract

**Highlights:**

**Public health relevance—How does this work relate to a public health issue?**

- Young adults with advanced cancer have distinct psychosocial and existential care needs with limited access to age-relevant support.
- Nature-based interventions represent a promising but underexplored area of supportive care for this population.

**Public health significance—Why is this work of significance to public health?**

- The findings suggest that nature-based rehabilitative palliation may provide meaningful support for young adults with advanced cancer.
- The study highlights the importance of capturing situational benefits in palliative intervention research.

**Public health implications—What are the key implications or messages for practitioners, policy makers and/or researchers in public health?**

- Nature-based rehabilitative palliation may complement existing support services by providing opportunities for distress relief and peer support.

**Abstract:**

Background/Objectives: Young adults living with advanced cancer often experience substantial psychological and existential health challenges, while frequently being underserved by existing rehabilitative and palliative services. This mixed-methods study evaluated the effects and experiences of a nature-based intervention designed to ease the burden of distress and support coping in the context of an incurable illness. Methods: Four consecutive intervention groups participated in a six-week program consisting of one overnight nature camp and three outdoor sessions. Quantitative data on quality of life (FACT-G) were collected before and after each intervention cycle. At the session level, pre–post measures assessed distress, vitality, resilience, and perceived connectedness. Qualitative data consisted of post-intervention semi-structured group interviews. Results: Twenty-four participants completed the intervention. No significant change was observed in overall quality of life which is in line with expectations for palliative interventions for individuals with incurable illness. In contrast, session-level analyses showed consistent reductions in distress and increases in vitality, resilience, and connectedness across multiple domains. The qualitative findings indicated that the intervention provided a supportive context for emotional regulation, coping, social belonging and existential orientation. Conclusions: Together, the findings support that nature-based rehabilitative palliation may offer meaningful support for young adults living with advanced cancer. The study underscores the importance of multi-level mixed-methods evaluation for capturing the value of palliative interventions where temporality and situational benefit constitute central outcomes.

## 1. Introduction

### 1.1. Living with Cancer

Patients with advanced cancer are now living longer due to advances in oncologic and hematologic therapies [1]. Yet the psychological and existential challenges of living with cancer, alongside the physical burden, are often overlooked [1,2]. Based on 2022 estimates, 20 million people are diagnosed with cancer globally every year, with young adults (aged 25–49 years) accounting for approximately 7% of new cases [3].

Being diagnosed with cancer as a young adult, at a life stage where identity and future aspirations are still taking shape, can cause immense psychological distress, including challenges to identity and loss of meaning [4,5]. Living with advanced cancer compounds this burden further, as facing one’s own mortality and a limited lifespan raises existential challenges that extend beyond those associated with curative treatment [6,7]. Although this burden cannot be eliminated, it can be lessened through appropriate interventions that support coping, community, and quality of life [7,8].

In Denmark, psychosocial cancer support and rehabilitation are primarily delivered through municipalities and the Danish Cancer Society. However, younger adults frequently decline municipal rehabilitation services [8], where the majority of participants are above 60 years of age. Consequently, many young adults do not receive adequate support in navigating their complex life situation. The present intervention at the Copenhagen Centre for Cancer and Health, Denmark, was therefore developed to address the needs of this small but very vulnerable group in the Capital Region of Denmark and the Region of Zealand. Together, these regions comprise approximately 2.8 million inhabitants [9] and account for around 20,600 new cancer cases and 7100 cancer-related deaths annually [10]. The proportion of young adults living with advanced cancer in these regions, the focus of the present project, is not reported. However approximately 600 new cases of cancer are diagnosed each year among young adults in the Capital Region alone [11].

### 1.2. Natural Environments as a Health Resource

In recent decades, nature-based interventions have received increasing attention due to growing evidence of their health benefits. The term encompasses an umbrella of structured approaches that utilize exposure to and interaction with natural environments to promote health and well-being [12]. A growing body of research demonstrates significant positive effects across emotional, cognitive, and physiological domains, including reduced anxiety, depression and stress, and improved overall well-being [12,13,14], although the pathways underlying these effects are not yet fully understood.

Natural settings that offer serenity, biodiversity, and refuge have been associated with stress reduction and improved mood [15]. These outcomes are often attributed either to pleasant and varied sensory stimulation that encourages attentional soft fascination thereby reducing cognitive load and rumination [16], or to evolutionarily shaped preferences for natural environments associated with survival [17]. From a social perspective, nature-based settings appear to facilitate group processes differently than indoor spaces. Studies indicate that outdoor environments may enhance social interaction and group cohesion, as well as participants’ openness and willingness to engage in therapeutic group conversations [18,19], potentially due to an atmosphere perceived as less restrictive than indoor clinical settings.

A more recent line of inquiry has focused on the construct of nature connection, understood as the subjective sense of closeness with the natural world [20]. Research indicates that exposure to nature can enhance this sense of connectedness, which in turn is associated with increased psychological well-being [21]. Within this field of research, an existential dimension has been identified, often referred to as eudaimonic well-being, relating to how encounters with nature can support reflections on meaning, identity, and one’s place within a larger ecological context [22,23,24]. This dimension shifts the focus from immediate psychological benefits (hedonic well-being) toward how engaging with the natural world can serve as a context for navigating existential concerns.

### 1.3. Nature Interventions for Young Adults with Cancer

Although literature remains limited, emerging studies suggest that nature-based interventions may benefit people living with cancer, including young adults [25,26], with findings indicating reductions in anxiety, depression and stress, as well as improvements in coping and mental well-being. In a study by Timko Olson et al. [27], young adults with cancer describe nature not only as a resource for emotional support but also as a meaningful context for reflection on identity and meaning that helps them situate their illness experience within a broader life narrative. This engagement extends beyond alleviation of mental distress, positioning nature as an active resource for navigating existential challenges, and aligns with the eudaimonic dimension of nature connection mentioned above.

While no research has yet examined nature-based interventions specifically for young adults with advanced cancer, the existing evidence on young adult cancer survivors suggests that such interventions may also hold potential for lessening the psychosocial and existential burdens experienced by this particularly vulnerable group [25,26,27]. The eudaimonic aspects of nature connection are especially relevant here, as individuals living with incurable illness often face existential challenges related to identity, meaning, and mortality [6,7], challenges for which nature may offer a meaningful and supportive context [27].

Drawing on the existing evidence base and nature-based pilot programs at the Copenhagen Centre for Cancer and Health [28,29], combined with the expressed needs of young adults [8], a method for nature-based rehabilitative palliation was developed at the center. Outcomes from the pilot programs were notably positive: participants described that the nature-based activities and shared experiences in a peer community lessened the burden of mental distress and provided sources of joy and meaning. These preliminary results warranted further study, leading to the present study evaluating a nature-based intervention delivered to four consecutive groups of young adults with advanced cancer conducted by the Copenhagen Centre for Cancer and Health.

### 1.4. Aim

The present study evaluates a nature-based intervention for young adults with advanced cancer, aiming to ease the burden of psychological distress and support participants in navigating life challenges. The effect of the intervention is assessed across several outcomes, including mental distress, resilience, vitality and connectedness, alongside participants’ self-reported experiences and perceived benefits of participation.

## 2. Materials and Methods

### 2.1. Study Design and Sample Size

The study used a mixed-method design with questionnaires and group interviews. This approach allowed for the integration of quantitative outcomes with participants’ experiential accounts, enabling both the assessment of effects and a deeper understanding of underlying processes. The intervention was delivered to four groups between the spring of 2025 and the spring of 2026. Each group completed a 6-week program comprising two 3 h sessions, one 26 h session, followed by an additional 3-h session. The content of the intervention is described below. Data were collected at baseline and immediately after each intervention cycle, as well as before and after each individual intervention session. An a priori power analysis was conducted using G*Power 3.1 (Heinrich Heine University Düsseldorf, Germany) based on a paired-samples *t*-test approximation (two-tailed α = 0.05, power = 0.80). Assuming a moderate-to-large effect size, a minimum sample size of 26 participants was required. The effect-size estimate was informed by findings from the preceding pilot program [29], in which positive changes were observed in immediate psychosocial outcomes following nature-based sessions. Given the small and difficult-to-reach population of young adults living with advanced cancer, the study was designed as an exploratory investigation of feasibility and potential intervention effects and was not powered to detect small effects. To allow for attrition, the recruitment target was aimed at 40 participants.

### 2.2. Recruitment

Recruitment was conducted on an ongoing basis from autumn 2024 to late winter 2025. The opportunity to participate in the study was communicated through several channels. The primary recruitment pathway was through staff at the Copenhagen Centre for Cancer and Health. Oncological and hematological departments at hospitals in the Capital Region of Denmark and the Region of Zealand were also informed about the study, and they helped disseminate written information about participation. Likewise, the Danish Cancer Society disseminated information about how to participate via social media. Potential participants were encouraged to self-refer by contacting a staff member at the Copenhagen Centre for Cancer and Health, which was responsible for delivering the intervention. Informed consent was obtained from all participants following the guidelines of the Danish National Research Ethical Committee.

### 2.3. Inclusion

At the initial contact, potential participants were informed about the study and the nature-based intervention. Those expressing interest were invited to a subsequent consultation, during which the healthcare professionals assessed their expectations, needs and resources, and screened for eligibility based on the inclusion criteria. Eligibility criteria were as follows: advanced cancer, defined as a malignancy not expected to achieve permanent remission with currently available treatments, although prolonged survival may be achievable [30]; age between 25 and 45 years; sufficient Danish language proficiency; and a moderate level of physical function, defined as independence in activities of daily living and the ability to walk 1.5 km at a slow pace. Exclusion criteria were self-reported substance abuse or a medical recommendation to avoid outdoor activities and/or social interaction.

### 2.4. The Nature-Based Intervention

The intervention draws on adventure- and nature-based therapy [31,32,33] and integrates elements from cancer rehabilitation and palliation [34], especially social and mental elements. The approach has a salutogenetic perspective in which illness is not the central focus, but where attention is directed toward resources [35]. Through an interaction between the core elements of sensory experiences, skill building, nature narratives and knowledge, mirroring, and symbols, the healthcare professionals sought to facilitate experiences of relief from distress, reflection, and connectedness.

Nature-based experiences and activities with peers, such as walking in the forest and seeking patterns in nature, were used to foster momentary relief and joy. These experiences also provided coping strategies for stress relief that participants could transfer to everyday life. Skill-building activities, such as lighting a fire with a fire rod or constructing a hammock, were used to broaden participants’ sense of self beyond their illness by fostering agency. Nature knowledge and narratives were used to offer perspective, providing a lens through which participants could reflect on life’s sometimes harsh conditions. In this process meaning may arise and joy and acceptance can be rediscovered.

The three-hour sessions took place in a natural environment at a campsite equipped to meet participants’ basic needs, including warmth, shelter, adequate nourishment, and toilet facilities, providing a foundation for healthy outdoor engagement [32]. The site is close to public transportation and parking. The overnight camp took place in a secluded and serene natural environment surrounded by forest and a meadow, with indoor cooking, sleeping and toilet facilities (a cabin).

#### 2.4.1. Content of the Sessions

Each session began by fostering calmness and a sense of safety within both the natural environment and the group through meditative, sensory-based experiences. More active, aesthetic, and emotional forms of engagement were gradually introduced, including bushcraft and artistically expressive activities alongside nature narratives. Session content was continuously adjusted in response to weather conditions and participants’ day-to-day variations in physical and mental functioning.

The three-hour sessions followed an established structure. Participants arrived at the parking area and bus stop and walked to the site together, using the journey as an informal walk-and-talk activity. Upon arrival, participants were welcomed with a hot drink by the fire and a presentation of the program. A grounding sensory activity aimed at calming the nervous system was then guided by one of the nature therapists, followed by nature-related storytelling, a bushcraft or creative activity, and a shared meal by the fire. Each session concluded with group reflections on experiences and personal insights. The session themes and main activities are presented in the table below (Table 1).

#### 2.4.2. Staff

The intervention was delivered by two staff members, a dietitian and an occupational therapist, both holding professional healthcare qualifications, substantial experience in cancer rehabilitation and palliation, and a master’s degree in Nature-Based Therapy and Health Promotion from the University of Copenhagen. The overnight camp was additionally staffed by a cancer nurse. All staff were organizationally embedded within the Copenhagen Centre for Cancer and Health.

### 2.5. Quantitative Data

#### 2.5.1. Pre–Post Intervention Data

Before each intervention cycle, participants completed a baseline questionnaire covering sociodemographic characteristics, diagnosis, previous and current engagement with rehabilitation services, and use of nature. Before and after each intervention cycle, participants completed the validated questionnaire Functional Assessment of Cancer Therapy-General (FACT-G), which measures self-reported quality of life among individuals with cancer [36]. The questionnaire consists of 27 items rated on a 5-point Likert scale and is distributed across four domains: physical, emotional, social, and functional well-being. Total scores range from 0 to 108, with higher scores indicating better quality of life.

#### 2.5.2. Pre–Post Session Data

Before and after each session (the overnight camp and the three shorter sessions), participants completed a short two-part online questionnaire comprising single-item Likert-scale measures designed to minimize participant burden while capturing changes in participants’ immediate mental states. The first part assessed three single-item measures related to distress, vitality, and resilience. Questions were adapted to capture participants’ immediate states for each measure to examine whether the session had direct influence on these parameters. Distress was measured using a single item adapted from the Distress Thermometer, a brief screening instrument for assessing psychological burden in individuals with cancer [37]: “Please mark the number between 0 and 10 that describes your overall experience of distress right now.” Responses were rated on an 11-point Likert scale ranging from 0 (no mental distress) to 10 (maximum mental distress). Vitality was measured using a single item adapted from the Subjective Vitality Scale [38]: “At the moment I feel alive and full of vitality.” Responses were rated on a 7-point Likert scale ranging from 1 (completely disagree) to 7 (completely agree). Resilience was measured using a single item adapted from the State-Trait Assessment Resilience Scale [39]: “At this moment, I feel capable of dealing with potential difficulties,” rated on a 5-point Likert scale ranging from 1 (completely disagree) to 5 (completely agree).

The second part of the online questionnaire assessed momentary sense of connectedness across five domains: nature, oneself, other people, the present moment, and something greater in life. The first domain corresponds to the construct of nature connection, defined as the subjective sense of closeness to the natural world [40]. The remaining four domains correspond to the dimension of connectedness within existential and spiritual health [41,42].

Each domain was measured using a single item adapted from the Inclusion of Nature in the Self Scale [40] that was formulated as a reflection: “Consider which image best reflects how connected you feel to [domain] right now.” Each reflection was accompanied by a visual representation featuring seven pairs of circles with progressively increasing overlap, representing varying levels of perceived connectedness. Responses were rated on a 7-point Likert scale ranging from 1 (low perceived connectedness) to 7 (high perceived connectedness).

#### 2.5.3. Quantitative Analysis

Questionnaire data were analyzed per protocol, including only participants who completed the intervention (treatment-on-the-treated; TOT). As the questionnaires were administered online and required responses to all items for submission, no item-level missing data were present. Participants with missing pre- or post-data were excluded from the relevant comparison. The normal distribution of the pre–post intervention data was evaluated using the Shapiro–Wilk test.

### 2.6. Qualitative Data and Analysis

Semi-structured group interviews were conducted at the Centre for Cancer and Health within one week of each intervention cycle. The staff responsible for conducting the intervention were not present at the interviews. The interviews lasted approximately 1.5–2 h and followed a semi-structured interview guide focusing on participants’ reflections on being in nature, participating in a peer group, and engaging in the guided activities (Supplementary Materials). With participants’ permission, the interviews were audio-recorded and subsequently transcribed. The qualitative analysis followed a reflexive thematic analysis approach, as described by Braun and Clarke [43]. The process involved iterative cycles of familiarization with the data through listening to the recordings and repeated reading of the transcripts, conducted alongside initial coding. The analysis then progressed through refinement of codes and the development and review of themes. The analysis was mainly carried out by the first and last authors, who were not involved in delivering the intervention. The first author conducted the coding and initial development of themes, while the last author contributed through ongoing analytic discussions and reflexive review of emerging codes and themes. To enhance the trustworthiness of the analysis, transparency, reflexivity, and coherence were emphasized throughout the analytic process [43,44]. Reflexivity was maintained through ongoing critical reflection on assumptions and analytic decisions, particularly during discussions between first and last authors, with all authors engaged in the finalization of the themes. Credibility was supported by close and sustained engagement with the data throughout the analysis and by grounding interpretations in data extracts. Differences in interpretation were addressed through dialogue and revisiting of the data.

## 3. Results

### 3.1. Participant Flow and Characteristics

**Figure 1:**
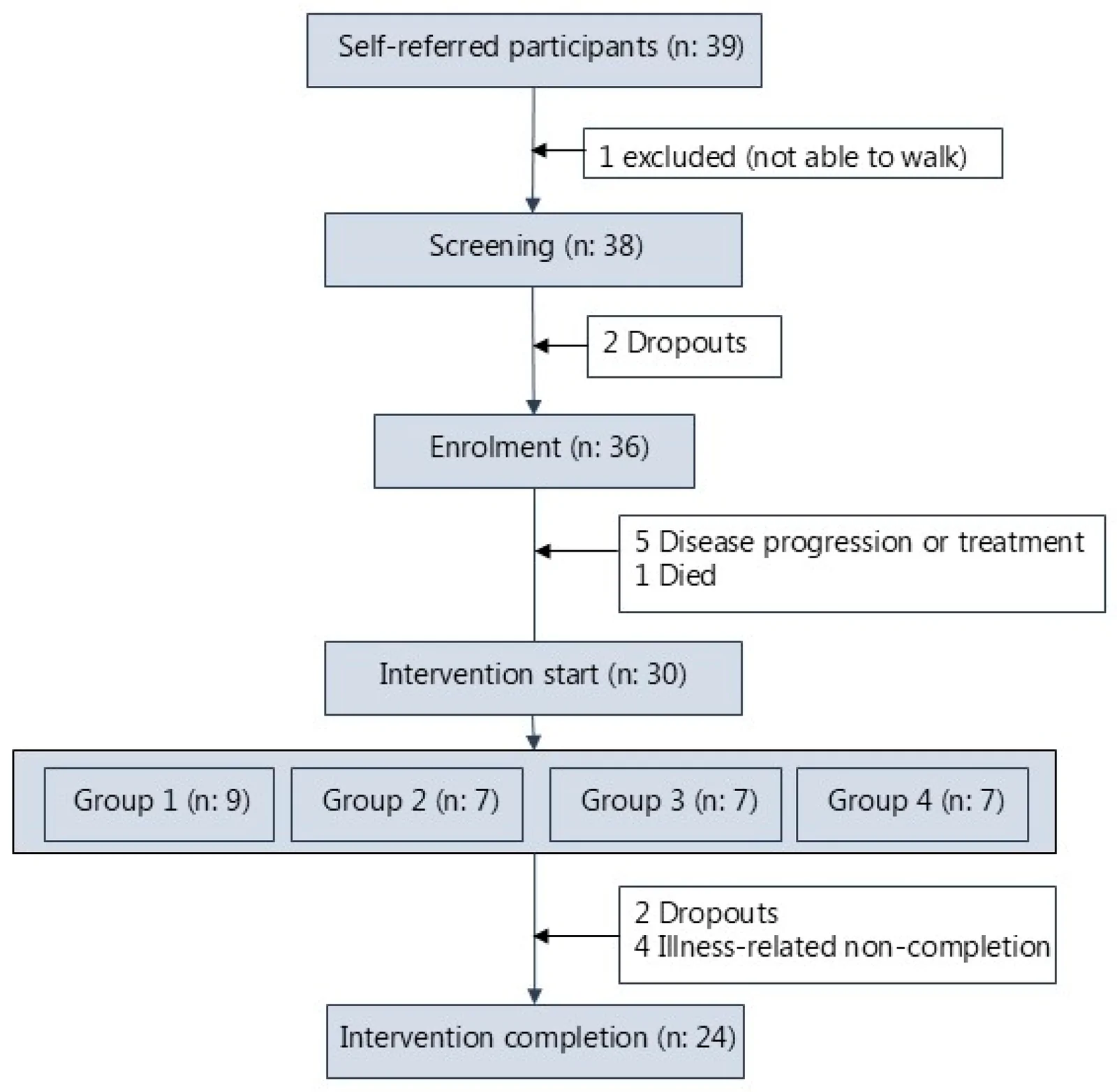
Participant flow.

Of the 39 potential participants, 38 met the eligibility criteria. Thirty commenced the intervention and 24 completed it (Figure 1). Completion was defined as participation in the overnight camp and at least one additional session, or attendance in all the shorter sessions. The primary reasons for not starting the intervention after being found eligible, non-completion, and absence from individual sessions were general illness, treatment appointments and disease progression. Four participants dropped out between the initial screening and intervention completion, citing limited capacity or change of mind.

Of the 24 participants who completed the intervention, 18 were female and 6 were male, with ages ranging from 30 to 45 years and an average age of 39 years. The most common diagnosis was breast cancer followed by lung and brain cancer. Although eligibility criteria included adults from the age of 25, no individuals from the 25–29 age group self-referred. The present study and its findings therefore primarily reflect the experiences of individuals with advanced cancer in the later phase of young adulthood.

Participants reported on their use of natural environments in everyday life. The majority were frequent users, with visits occurring daily to weekly, suggesting that most participants already had an affinity for spending time in natural environments.

### 3.2. Quantitative Results

#### 3.2.1. Pre–Post Intervention Results

The Shapiro–Wilk test showed no significant deviation from normality (*W* = 0.92, *p* = 0.73) and differences between pre–post measures were assessed using a paired-samples *t*-test. No statistically significant difference was observed in the Functional Assessment of Cancer Therapy (FACT-G) mean score between baseline and post-intervention (*p* > 0.05; Table 2), indicating that participants’ health-related quality of life did not change over the course of the intervention, although the interpretability is limited due to the small sample size.

Participants’ mean scores indicate a moderate level of quality of life, consistent with levels commonly reported among cancer patients undergoing treatment or living with advanced disease, and below general population norms [45].

#### 3.2.2. Pre–Post Session Results

A paired Wilcoxon signed-rank test, suitable for ordinal Likert-scale data [46], was applied to examine pre–post differences in single-item outcomes at the session level. The results showed a significant decrease in distress from pre- to post-session with effect sizes in the large range [47] (*p* < 0.001, r > 0.50; Table 3). Inspection of paired responses revealed decreases from pre- to post-measurements in 72 of the 82 observations for distress. Significant pre–post differences were also observed for vitality (*p* < 0.001) and resilience (*p* < 0.001), both of which showed large increases in effect sizes (r > 0.50). These results should be interpreted with caution due to the ordinal nature of the Likert-scale data and the limited interpretability of change magnitude. However, the consistency of pre–post change across participants and sessions supports the robustness of the observed effects. Direct comparisons between the three scales are not appropriate due to differences in scale design.

Momentary sense of connectedness to nature, oneself, other people, the present moment, and something greater in life showed significant increases across all measures from pre- to post-session with relatively large effect sizes (*p* < 0.001, r > 0.50). The largest increase in mean score was observed in connectedness to nature, followed by connectedness to others and to oneself (Table 4). Visual inspection of paired responses showed increases in the majority of observations. As noted above, these results should be interpreted with caution due to the ordinal nature of the Likert-scale data and the relatively small sample size; however, the consistency of the observed effects across participants and sessions supports the robustness of findings.

### 3.3. Qualitative Results

Across the four group interviews, with a total of 16 young adults participating, four overarching and interconnected themes were identified: (1) A refuge for relief and coping; (2) A shared focal point in a caring context; (3) Community and reduced loneliness; and (4) Existential orientation and perspective. These themes appeared consistently across groups, demonstrating substantial thematic convergence with variation at the individual level. Although intertwined in practice, the themes are presented individually below for analytical transparency.

#### 3.3.1. A Refuge for Relief and Coping

Across group interviews, participants described how the sessions in nature were experienced as a restorative refuge in which both body and mind could settle, allowing illness- and treatment-related burdens to temporarily recede into the background. This created space for positive emotional experiences, including immediate joy, vitality, and gratitude, as well as a sense of increased capacity to engage with personal challenges. Two participants articulated this experience as follows:

*“These are the only moments, since I received my diagnosis, where I actually feel that I can breathe freely, and I find that quite remarkable.”*

(PA16)

*“Just experiencing the energy that starts to build up and getting more joy and more resources and more of a sort of desire to deal with everyday life.”*

(PA08)

Several participants pointed out that the guided sensory facilitation made it easier to be present and to take in nature’s sensory impressions, thereby enabling a sense of presence among participants who otherwise found it difficult to be in their bodies due to pain, anxiety, or restlessness:

*“I could not tolerate being in my own body, because I could feel pain and anxiety and everything. So there was some need for acclimatization […]. But this thing, that you can be guided in this way, I really think that has been a great help.”*

(PA07)

Participants furthermore described how the positive emotional regulation extended beyond the session itself. For some, this led to more sustained changes, whereas for others it was closely linked to attending the sessions. Two participants described the after-effects as follows:

*“You think differently and get a completely different feeling in your body when you get home* [after a session]*.”*

(PA04)

*“After this* [the intervention]*, I have had a different kind of calm and felt relieved in some way.”*

(PA09)

The sessions were not only experienced as a rare respite from the continual presence of distress, but also as a context in which the fragmented sense of self, associated with being reduced to a patient identity, was temporarily diminished:

*“And what I then experienced, when I went into nature, was actually that I began to feel whole again.”*

(PA05)

The alleviating effect did not lie in symptom reduction, but rather in a temporary shift in the perceived weight of the burden, enhancing participants’ mental capacity to bear it:

*“My pain is still there, but maybe I can handle it better after a day like that.”*

(PA16)

Taken together, these findings indicate that the intervention supports affective and physiological regulation, whereby nature’s potential for calm and relief becomes more apparent and accessible through facilitation. This is consistent with existing research on nature-based interventions for individuals with cancer [25,26,27]. For some participants the effects extend beyond the session itself, enhancing their resources for coping with ongoing challenges.

#### 3.3.2. A Shared Focal Point in a Caring Context

A central finding is that nature functioned as a shared focal point, a common third, which together with the salutogenic focus of the sessions, contributed to an interaction that was not centered on illness, but rather on shared nature-based activities, sensory engagement, and social interaction. Participants described this as a decisive difference from other forms of support:

*“There was a third focus. We had not gone out there because we were expected to talk about being sad. It was not about illness, although it was present in the background.”*

(PA03)

At the same time, learning new skills and rediscovering one’s own capabilities seem to have strengthened the experience of a more coherent self:

*“It also gives you something like, wow, I can do this too, I am not just ill.”*

(PA15)

Illness was thus not absent, but neither was it dominant. This context allowed conversations to arise and fade naturally, without demands for continuous participation. Silence was accepted as a legitimate part of being together:

*“I think the right place is in nature. Yes. Because you have different kinds of conversations and that sense of being part of something bigger, and things like that. It does not feel so awkward, if I can put it that way.”*

(PA14)

The outdoor setting, across all weather and seasons, inherently created a caring environment in which therapists attended to participants’ well-being throughout the sessions. Experiences of care are consistently highlighted as a significant element, particularly the bodily and relational dimensions of care. Several participants describe the experience of being looked after by the therapists as both unfamiliar and emotionally meaningful:

*“Being taken care of so well, even though you are an adult, that can really move me.”*

(PA01)

*“The greatly increased care that we could all feel* […] *has helped to increase—at least from my perspective, well-being in my own body and has contributed to greater self-care.”*

(PA07)

The sense of care, or of being “held” in a difficult life situation, was also related to nature itself:

*“You feel like you have a big brother who looks after you. And that is, in a way, the feeling I have with nature. As if I have something I can lean into a bit, something that can catch me when everything feels difficult.”*

(PA02)

This holistic experience of care stands in contrast to the more standardized forms of care commonly encountered in the healthcare system, which, due to its necessary focus on disease, may also contribute to a fragmentation of self-understanding when illness occupies a substantial part of everyday life. The nature context and activities seemed to contribute to a more holistic self-concept that transcended illness identity. This perspective aligns with the concept of existential health [48], in which relational safety, dignity, and the experience of being more than one’s illness are highlighted as central dimensions, particularly in palliative contexts [42,48].

#### 3.3.3. Community and Reduced Loneliness

The composition of the group, young adults living with advanced cancer, was consistently highlighted as fundamental to both the motivation to participate and the perceived benefits of participation, alongside the nature setting itself. Particularly significant was the experience of being together with others who shared a similar life situation. Several participants described the relief of shared recognition:

*“There are also others who are going through the same as me. So just not necessarily having to talk about everything but simply being there together with some others. That unspoken community we have, it provides a kind of safety, in a somewhat strange way.”*

(PA03)

This sense of recognition was directly linked to a reduced experience of loneliness:

*“You do not feel alone in the loneliness or in the situation.”*

(PA15)

At the same time, being part of a community with peers also raised ambivalence. Encountering others’ illness can be challenging, and several participants emphasized that participation requires a certain degree of readiness:

*“And this is the thing, when we talk about the group being important, that recognition that is there can also be hard.”*

(PA01)

*“One needs to have some kind of psychological robustness. You have to be ready to meet yourself.”*

(PA13)

The findings suggest that even though community within a shared life situation of advanced cancer in young adulthood primarily functions as a source of support, it also constitutes a potential burden. Analytically, the experience of relief can be understood in relation to the concept of existential loneliness, which recent research has identified as a central condition in serious illness [49]. Studies indicate that communities of others in similar life situations can reduce such loneliness by diminishing the need for explanation and emotional filtering, while at the same time potentially being psychologically demanding [50], as is also reflected in the participants’ accounts.

#### 3.3.4. Existential Orientation and Perspective

Participants articulated, in various ways, how changes in nature served as a mirror through which they gained renewed perspectives on their own situation. These reflections and shifts were often described as subtle and experiential:

*“Changes happen, but there are also things that persist, and you are made to withstand and go through things. That is something that stays with me now, without me really thinking about it. Yes, it is very difficult to explain.”*

(PA16)

A recurring theme in the participants’ accounts was the experience of being part of something larger than themselves:

*“Whether you look up or down, you feel that you are part of something bigger.”*

(PA13)

It is evident that being in nature and participating in the sessions affected several participants at a profound level that was challenging to verbalize, suggesting these experiences exceed what language can readily capture:

*“I actually think that something awakened a small depth in me, which I can tend to forget. But something you only discover when you are in complete calm.”*

(PA01)

*“It has helped to increase that sense of joy –being in my own body, but also life itself. Now I know this is a little bit existential. But I also think that is an important point. That has been, at least, what mattered most to me.”*

(PA8)

These experiences point to an existential dimension in the participants’ accounts, in which meaning and identity are placed in a broader perspective that also provides relief and meaning. As mentioned in the Section 1, research on nature connectedness has demonstrated that contact with nature can also affect eudaimonic dimensions such as meaning and perspective expansion [22,24], which the participants’ experiences corroborate. In this context, nature appears as a bodily grounded space for reflection, where existential perspectives can emerge as lived sensory-based experiences rather than through explicit verbal processing.

As one participant succinctly expressed it:

*“This is really an injection of meaning into life”.*

(PA10)

## 4. Discussion

The present study evaluated a nature-based intervention for young adults living with advanced cancer, focusing on easing the burden of psychological distress and providing resources for coping with life challenges. A central perspective emerging from the findings is the situational nature of the outcomes. The absence of change in overall quality of life is consistent with research on palliative interventions for advanced cancer [51], where stability rather than improvement in global outcomes is regarded as compatible with the aims of palliation. This finding should, however, be interpreted with some caution, as the relatively small sample size may have limited the ability to detect more modest changes. At the same time, palliative research supports that such changes are limited in a population facing severe and progressive illness [51,52], as reflected in the severity of participants’ condition, with six participants dying within three months of completing the intervention.

In contrast to the global outcomes, the session-level quantitative results revealed consistent state-based reductions in distress and increases in vitality and resilience, which were supported by the qualitative findings where the participants described a sense of calm and relief with a temporary shift away from illness-related concerns. Together, these results suggest that the intervention’s effects on relief and coping primarily operate at the level of state-based processes. This aligns with theory and research on coping with serious illness, which emphasizes the importance of repeated experiences of emotional regulation and relief in the context of persistent and non-modifiable stressors [2,53]. From this perspective, palliation in the context of advanced cancer may be understood as facilitating conditions for situated meaningful experiences that can be revisited over time, rather than enduring changes. This has methodological implications. By combining quantitative measures of immediate change with qualitative data, it is possible to examine both observed changes and participants’ accounts of these changes to gain understanding of the underlying processes. The findings altogether therefore support the use of mixed-method and multi-level approaches in the evaluation of palliative interventions, where relevant outcomes may be situational and not fully captured by global measures. This perspective further invites a reframing of how value is conceptualized. Temporality may be understood as an intrinsic condition of meaningful support in the context of incurable illness, where the opportunity to access moments of relief, connection, and perspective may represent a central contribution of such supportive interventions.

### 4.1. The Natural Environment as an Enabling Context

A central component of the intervention is the natural environment, which appeared to function as a co-constitutive element in shaping the observed outcomes. As outlined in the introduction, exposure to natural environments has been consistently associated with stress reduction, emotional regulation, and attentional restoration [12,13,14]. The session-level reductions in distress and concurrent increases in vitality observed in this study are compatible with these mechanisms and reflected in the qualitative findings, where participants describe the environment as calm, supportive, and conducive to a sense of presence which suggests that the nature-based setting supports immediate bodily and emotional regulation in this vulnerable population too. The interview findings further indicate that these regulatory effects are strengthened through therapeutic guidance and attentive care, which helped participants attune to sensory experiences and remain present despite physical or psychological strain. In line with previous research, the outdoor natural setting also appeared to influence the conditions under which social interaction unfolded, indicating that therapeutic processes may be facilitated differently in outdoor contexts compared with indoor settings [18,19]. In the present study, the qualitative findings suggest that shared engagement with nature and nature-based activities can provide a common focus and foster a sense of agency. Which, in turn, allows a more coherent sense of self to emerge momentarily, offering relief from the fragmented self-experience often associated with advanced illness [6,7].

### 4.2. Connected by Nature

The session-level increases in connectedness across multiple domains represent a consistent finding. These findings are reflected in the qualitative data, where participants describe experiences related to belonging, reduced loneliness, and enhanced sense of oneself. The peer-based community, in which illness narratives were not positioned at the center, was described as alleviating feelings of existential loneliness associated with living with an incurable illness [49], consistent with the increased sense of social connectedness observed in the session-level outcomes. Engagement with the natural environment, in combination with the structured activities, also seemed to facilitate subtle shifts in perspective, whereby participants’ understandings of themselves and their lives were situated within a broader context. Observations of natural processes and cyclical changes, supported by nature-based narratives, were described as particularly important in enabling these shifts. This aligns with research on the eudaimonic dimensions of nature connectedness, which emphasizes meaning and perspective expansion as outcomes [22,23,24]. These findings resonate with broader conceptualizations of existential health, in which meaning, identity, and connection are regarded as central dimensions [48]. Being in a natural environment with peers and therapeutic guidance thus appears not only to enhance state-based aspects of well-being related to emotional regulation, but also to provide situational support for existential dimensions of health through multiple forms of connectedness. This may hold particular relevance for individuals navigating the existential challenges associated with incurable illness and limited lifespan.

Overall, the outcomes support the adequacy of the nature-based intervention content and its core elements—sensory experiences, skill building, nature narratives and knowledge, mirroring, and symbols—in facilitating momentary relief from distress, enhancing resources for coping and addressing existential concerns.

### 4.3. Methodological Considerations and Limitations

A limitation of the study is the relatively small sample size, slightly below the target size identified, which limits the ability to detect small changes, particularly in global measures such as FACT-G, though also not expected in palliative interventions. This should however be considered when interpreting the non-significant findings in the global outcome. However, the observation of consistent large effects in session-level outcomes supports the sensitivity of the study to detect changes of greater magnitude.

Methodologically, the divergence between intervention-level and session-level outcomes as well as the complex and highly challenging life situation of having an advanced illness highlights the importance of multi-level mixed-methods assessment as mentioned above. A limitation of the state-based Likert measures lies in their restricted construct validity, as complex and nuanced experiences are reduced to single numerical judgments. At the same time, their strengths include sensitivity to immediate subjective mental states and low cognitive demands. This points to the need for further development and testing of appropriate research tools to capture state-based changes in the realm of continuous adverse life circumstances.

The interpretation and generalizability of the findings are subject to several limitations. Participation in the study was based on self-referral, which may have introduced selection bias, as individuals with a particular interest in, or openness toward, nature-based approaches may have been more likely to enroll. The sample consisted predominantly of women, which limits the transferability of the findings to young adult men living with advanced cancer. Although the inclusion criteria encompassed young adulthood broadly, no individuals from the 25–29 age group were represented, and participants were mainly in the later phase of young adulthood. Furthermore, the small sample limits the generalizability of the conclusions. To reduce potential response bias in data collection, quantitative data were collected via online questionnaires.

### 4.4. Implications for Practice and Future Research

The present study provides initial support for the potential of nature-based interventions as a complement to existing rehabilitative and palliative services for young adults living with advanced cancer. As the intervention was developed and delivered within an existing cancer rehabilitation and health promotion center, the approach appears feasible to integrate within established rehabilitation and palliative care pathways. Given the relatively small size of this population, implementation may be best achieved through structured collaboration between cancer rehabilitation centers, oncological and hematological departments, and regional or national cancer support programs, allowing participants to be recruited across a wider geographical area while maintaining age-specific peer communities. In such contexts, shorter intervention formats may be challenging to deliver due to travel distances, whereas residential formats such as overnight camps may represent a particularly feasible format. Further implementation should be accompanied by systematic evaluation. The situational and state-based nature of the observed outcomes highlights the importance of using evaluation designs capable of capturing immediate as well as longer-term effects. Future studies should therefore continue to apply multi-level and mixed-method approaches. To strengthen the evidence base, future research would benefit from including larger samples and recruiting a broader range of participants, including younger adults and a more balanced gender distribution. While inclusion of comparison groups would strengthen causal inference, withholding access to participation in the context of palliative and supportive interventions for individuals living with advanced illness may raise ethical concerns. Future research may therefore consider alternative study designs that allow for comparison while ensuring access to supportive care, such as comparative study designs. Finally, further studies may explore the transferability of the intervention to other populations with advanced illness, as well as the relative contribution of different intervention components, for example in relation to age and gender preferences.

## 5. Conclusions

This study evaluated a nature-based intervention for young adults living with advanced cancer, aiming to ease the burden of psychological distress and support participants in navigating life challenges. While no change was observed in global quality of life, consistent reductions in distress and increases in vitality, resilience, and connectedness were identified at the session level. These effects were supported by participants’ self-reported experiences, highlighting emotional regulation, social belonging, and existential orientation. Taken together, the results suggest that the intervention supports participants through state-based and experiential processes in finding temporary relief from distress and momentary support in the face of advanced illness. The results also point to the relevance of multi-level evaluation approaches in palliative research.

## Figures and Tables

**Table 1:** Sessions, themes and main activities.

Session	Duration	Theme	Main Activity
Session 1	3 h	Community	Lighting a fire
Session 2	3 h	Nature’s patterns	Building a hammock
Session 3	26 h	Nature’s cycles	Cooking over fire, sunrise meditation, forest walk
Session 4	3 h	Nature connection	Nature art

**Table 2:** Health-Related Quality of Life (n = 22).

Measure	Mean Pre	Mean Post	SD	*p* Value
FACT-G	71.18	72.32	10.16	0.605

**Table 3:** State-level experience of distress, vitality, and resilience, pre–post session (n = 82).

Measure	Mean Pre	Mean Post	SD	*p* Value	Effect Size *r*
Distress	5.43	3.21	2.08	*p* < 0.001	−0.79
Vitality	4.93	5.92	1.37	*p* < 0.001	0.69
Resilience	2.59	3.07	0.79	*p* < 0.001	0.72

**Table 4:** Sense of connectedness, pre–post session (n = 82).

Connectedness	Mean Pre	Mean Post	SD	*p* Value	Effect Size *r*
- Nature	3.96	5.55	0.94	<0.001	0.84
- Oneself	4.28	5.27	1.31	<0.001	0.69
- Others	3.87	5.04	1.28	<0.001	0.76
- Present moment	4.28	5.00	1.21	<0.001	0.68
- Something greater	3.84	4.73	1.28	<0.001	0.71

## Data Availability

Data are unavailable due to the General Data Protection Regulation restrictions on sharing. Permission to use the research data was solely granted to the named researchers involved in this study at the Faculty of Science, University of Copenhagen, Denmark.

## Supplementary Materials

The following supporting information can be downloaded at: https://www.mdpi.com/article/10.3390/ijerph23080953/s1, Table S1: Interview guide.
