# Supplementary material for: Connected by Nature: Nature-Based Intervention to Support Young Adults with Advanced Cancer
Source: Int J Environ Res Public Health. 2026 Jul 24;23(8):953. doi: 10.3390/ijerph23080953 (PMC13512662; doi:10.3390/ijerph23080953)
Supplement: Supplementary file 1 [file ijerph-23-00953-s001.zip › ijerph-4359338-supplementary.pdf]

Table S1. Interview guide.

Focus	Interview questions
Introduction	Thank you for agreeing to participate in this group interview about your experiences in the nature-based program. The interview focuses on your experiences, thoughts, and feelings during and after the program, and the significance it may have had for you.<br><br>I encourage you to engage in a conversation with each other about the questions I introduce. There are no right or wrong answers, and all perspectives are important. It is also completely fine to express critical views. You are welcome to respond to and build on each other's contributions.
Practical information, recording and anonymization	With your permission, the conversation will be audio recorded. The recording and transcription will be deleted once the study is completed. Until then, the data will be stored securely.<br><br>We will take a break halfway through. If you feel the need to move around, lie down, use the restroom, or anything else, please feel free to do so while we are talking.
Recalling significant experiences	To begin, we will sit quietly together for a moment. Try to recall the experiences, thoughts, and feelings you have had during and after the sessions in the nature-based program. Perhaps something stands out particularly strongly in your memory? You are welcome to write things down if that helps.
Initial questions about experiences	What have you taken away from participating in the program?<br><br>• What comes to mind spontaneously—feelings, memories, activities, relationships, insights?<br><br>• What stands out most strongly? Positively/negatively<br><br>• Are there still elements from the sessions you find useful in your everyday life or has anything changed for you in your everyday life?
Nature's influence/role	Has it had an importance to you that the sessions took place in nature?<br><br>• How did you experience being outdoors and in nature during the sessions, as opposed to, for example, being indoors or participating in other programs/activities?
Group dynamics	What role did being part of a group program play, if it played a role for you?

	• Was there anything about being with others in similar situations that influenced your experience—either positively or negatively?
Distress and development	Has participating in the individual sessions influenced your overall sense of distress? And if so, which way?<br>• Have you found yourself outside your comfort zone during the sessions?<br>• Have you experienced yourself in unexpected or new ways?<br>• What significance, if any, has this—and the program overall—had for you?
Transference to everyday life	Has your participation had an influence on your everyday life? If so, how?<br>• Are there any exercises you still use at home?<br>• Do you experience or use nature differently now, after the program, compared to before?
Program content and length	How did the practical aspects of the program/sessions work for you?<br>• What are your thoughts on the length, content, and structure?<br>• Was there anything you would have liked more of? Less of?<br>Concluding thoughts….
